# A case report of pregabalin misuse leading to drug dependence

**DOI:** 10.3389/fpsyt.2025.1511168

**Published:** 2025-03-21

**Authors:** Yaan Luo, Xiafeng Zhou, Ao Xiang, Yaqi Zeng, Jing Luo, Bojie Zhou, Nana Guo, Xuhui Zhou

**Affiliations:** ^1^ Department of Addiction Medicine, Hunan Institute of Mental Health, The School of Clinical Medicine, Hunan University of Chinese Medicine, Changsha, Hunan, China; ^2^ Department of Addiction Medicine, Hunan Institute of Mental Health, Brain Hospital of Hunan Province (The Second People’s Hospital of Hunan Province), Changsha, Hunan, China

**Keywords:** pregabalin, drug misuse, drug dependence, withdrawal symptoms, case report

## Abstract

Pregabalin misuse and dependence have become emerging concerns in recent years, particularly in regions where traditional drug-related crimes have been curbed, prompting users to seek alternative substances. Although pregabalin is primarily used for treating conditions such as epilepsy, neuropathic pain, and generalized anxiety disorder, its sedative and euphoric effects make it prone to misuse. This case report presents a 20-year-old male who developed pregabalin dependence after using the drug intermittently at escalating doses over a year. He experienced withdrawal symptoms including palpitations, tremors, irritability, insomnia, and auditory hallucinations upon cessation of the drug, which were alleviated by resuming pregabalin use. Upon admission, he was diagnosed with pregabalin dependence, hyperuricemia, and thyroid nodules. The patient underwent a comprehensive treatment plan involving benzodiazepines, antidepressants, and antipsychotics, leading to substantial improvement in mood, anxiety, psychotic symptoms, and withdrawal symptoms. This case highlights the growing issue of pregabalin misuse, the associated withdrawal symptoms, and the importance of early intervention and systematic treatment strategies. It emphasizes the need for stricter prescription controls, patient education on the risks of misuse, and multidisciplinary approaches to the treatment of pregabalin dependence. Further research is necessary to better understand the mechanisms behind pregabalin misuse and to develop improved prevention and treatment protocols.

## 1 Introduction

In recent years, as China has made strides in curbing traditional drug-related crimes, drug users have turned to alternatives, leading to the misuse of certain prescription narcotics and psychotropic drugs (1). Drugs that regulate GABA, including alcohol, benzodiazepines, and sleep medications, have been identified as having a high potential for misuse (2). Furthermore, prescription drugs once considered low-risk for misuse, such as gabapentin and pregabalin (collectively known as gabapentinoids), have also become targets of misuse. Though pregabalin does not directly bind to GABA receptors, it has a structure similar to γ-aminobutyric acid (GABA). Its higher potency, faster absorption, and greater bioavailability make pregabalin prone to misuse due to its sedative and euphoric effects (3). Users of pregabalin often exceed the recommended doses, seeking relaxation and pleasure, which can lead to significant health risks (4).

To date, no cases of pregabalin dependence have been reported in China. This case report presents a case of pregabalin misuse that led to dependence, providing a detailed analysis of the clinical manifestations, diagnostic processes, and treatment strategies for pregabalin dependence. This case aims to provide clinical evidence for the prevention and treatment of pregabalin dependence in the future.

## 2 Case

### 2.1 Case presentation

A 20-year-old male, without any significant prior medical history, was admitted to our hospital on September 3, 2024, for treatment of pregabalin dependence and withdrawal symptoms. The patient reported initiating pregabalin use in September 2023, when he began taking 75 mg tablets (brand name: Lyrica) purchased from abroad by the friend. Initially, he took 16–32 tablets daily, experiencing euphoria, slight body numbness, increased libido, and improved sleep. After a week, he noted diminishing effects and increased the dose to 80 tablets per day over the following month. Due to financial constraints, he began using pregabalin intermittently, although the exact dosage varied.

In December 2023, the patient learned of a more affordable domestic version of pregabalin (Xisiping, 75 mg) and switched to it, taking 32 tablets daily (family reported 64 tablets). Despite this adjustment, the patient continued to experience euphoria, energy, and improved sleep quality. In July 2024, the patient and his family attempted self-detoxification, ceasing pregabalin use for over 10 days. During this period, the patient experienced a range of withdrawal symptoms, including palpitations, fatigue, mild tremors, low mood, irritability, insomnia, and auditory hallucinations. He also displayed emotional instability, irritability, poor memory, and increased suspicion. He resumed taking pregabalin 32-64 tablets daily in August 2024 in an attempt to regain the euphoric effects, which alleviated his symptoms.

The patient had a history of smoking (one pack per day for seven years) and occasional cannabis use four years ago but had not relapsed. He denied concurrent use of any other psychoactive substances during pregabalin misuse. On admission, the patient’s vital signs were as follows: temperature 36.6°C, pulse 92 beats per minute, respiration rate 20 per minute, and blood pressure 113/64 mmHg.

### 2.2 Diagnostic evaluation

Upon admission, laboratory tests revealed no significant abnormalities in routine blood tests, coagulation function, myocardial enzymes, electrolytes, or thyroid function. Urine toxicology screen was negative for common drugs of abuse, including morphine, ketamine, ecstasy, methamphetamine, and buprenorphine. Liver function tests indicated low total protein (62.6 g/L) and elevated total bile acid (10.5 μmol/L). Kidney function tests showed elevated uric acid levels (510.9 μmol/L). A thyroid ultrasound revealed multiple cystic nodules in both thyroid lobes, categorized as TI-RADS category 2. Chest CT and brain MRI were unremarkable. An ECG indicated a normal sinus rhythm. The patient exhibited significant impairment in social functioning, unable to maintain normal academic and daily activities. No obvious abnormalities were found in physical and neurological examinations, and there was no evidence of any organic disease. The patient had no history of psychoactive substance use and no significant psychological stressors. Based on these findings, organic diseases, substance-induced mental disorders, severe stress reactions, and adjustment disorders can be temporarily excluded. The admission diagnosis was: Multiple drug and other psychoactive substance dependence syndrome, Hyperuricemia, Thyroid nodules.

Psychiatric assessment using the Hamilton Depression Rating Scale (HAMD), Hamilton Anxiety Rating Scale (HAMA), and Positive and Negative Syndrome Scale (PANSS) revealed significant depressive, anxious, and psychotic symptoms, with scores of 16, 25, and 98, respectively.

### 2.3 Treatment and clinical course

Detailed medication administration is provided in **Table 1**. Additional details regarding the treatment and clinical course are provided in **Supplementary Table S1**. Upon admission, the patient was started on intravenous drip diazepam (10 mg every 12 hours) for withdrawal symptoms, venlafaxine extended-release (75 mg daily) for depression and anxiety, and paliperidone extended-release (3 mg daily) for psychotic symptoms.

**Table 1:** Detailed medication administration.

	Day 1	Day 2	Day 3	Day 4	Day 5	Day 6
Diazepam	10mg Q12h IV	10mg Q12h IV	10mg Q12h IV	10mg Q12h IV	10mg Q12h IV	10mg Q12h IV
Venlafaxine	75mg Qd PO	75mg Qd PO	150mg Qd PO	150mg Qd PO	150mg Qd PO	150mg Qd PO
Paliperidone	3mg Qd PO	3mg Qd PO	3mg Qd PO	3mg Qd PO	3mg Qd PO	3mg Qd PO
Quetiapine			0.1g Qn PO	0.1g Qn PO	0.1g Qn PO	0.1g Qn PO
	Day 7	Day 8	Day 9	Day 10	Day 11	Day 12
Diazepam	10mg Qn IV	10mg Qn IV	10mg Qn IV	Discontinued		
Venlafaxine	225mg Qd PO	225mg Qd PO	225mg Qd PO	225mg Qd PO	225mg Qd PO	225mg Qd PO
Paliperidone	3mg Qd PO	3mg Qd PO	3mg Qd PO	3mg Qd PO	3mg Qd PO	3mg Qd PO
Quetiapine	0.1g Qn PO	0.1g Qn PO	0.1g Qn PO	0.1g Qn PO	0.1g Qn PO	0.1g Qn PO
Oxazepam				30mg Qn PO	30mg Qn PO	30mg Qn PO
	Day 13	Day 14	Day 15	Day 16	Day 17	Day 18
Diazepam						
Venlafaxine	225mg Qd PO	225mg Qd PO	225mg Qd PO	225mg Qd PO	225mg Qd PO	225mg Qd PO
Paliperidone	3mg Qd PO	3mg Qd PO	3mg Qd PO	3mg Qd PO	3mg Qd PO	3mg Qd PO
Quetiapine	0.1g Qn PO	0.1g Qn PO	0.1g Qn PO	0.1g Qn PO	0.1g Qn PO	0.1g Qn PO
Oxazepam	30mg Qn PO	30mg Qn PO	Discontinued			
	Day 19	Day 20	Day 21			
Diazepam						
Venlafaxine	225mg Qd PO	225mg Qd PO	225mg Qd PO			
Paliperidone	3mg Qd PO	3mg Qd PO	3mg Qd PO			
Quetiapine	0.1g Qn PO	0.1g Qn PO	0.1g Qn PO			
Oxazepam						

On day 3, the patient reported continued low mood, irritability, and insomnia, though he denied hallucinations or delusions. His psychiatric scales improved (HAMD 15, HAMA 24, PANSS 71). The venlafaxine dose was increased to 150 mg daily, and quetiapine fumarate (0.1 g nightly) was added to enhance sleep and manage residual psychotic symptoms.

By day 7, the patient’s sleep had improved, although he still felt fatigued and had low mood. His irritability had significantly decreased, with improved scores (HAMD 13, HAMA 15, PANSS 43). The venlafaxine dose was increased to 225 mg daily, and diazepam was reduced to 10 mg nightly.

By day 10, the patient reported no significant palpitations, tremors, or fatigue but still craved pregabalin. The evening dose of diazepam was discontinued, and oxazepam (30 mg nightly) was introduced. On day 15, the patient’s withdrawal symptoms had largely subsided, with HAMD and HAMA scores of 6 and 10, respectively. Oxazepam was discontinued.

On day 21, the patient reported normal sleep, stable mood, and reduced cravings for pregabalin, with no significant withdrawal symptoms or psychotic symptoms. The HAMD score improved to 3, and HAMA to 7. The patient requested discharge on September 25, 2024, and was continued on venlafaxine (225 mg daily) and paliperidone (3 mg nightly). No significant side effects were reported during the treatment.

## 3 Discussion

### 3.1 Pregabalin misuse

Pregabalin was approved by the U.S. Food and Drug Administration (FDA) in 2004 and introduced to China in 2010. It is widely used for treating adult epilepsy, neuropathic pain caused by diabetic neuropathy, postherpetic neuralgia, and fibromyalgia (5). The European Medicines Agency (EMA) also approved it for generalized anxiety disorder. Due to its sedative and anxiolytic effects, which are similar to those of benzodiazepines (6), pregabalin is prone to misuse. With the widespread clinical use of pregabalin, the trend of its misuse has also been on the rise (7). Analysis of the European Medicines Agency’s database reveals (8) that the number of reports of pregabalin abuse significantly increased from 2004 to 2014, indicating that the abuse of this drug has become an increasingly serious issue in Europe.

However, the U.S. Drug Enforcement Administration (DEA) classifies pregabalin as a Schedule V controlled substance, reflecting its lower potential for misuse compared to other controlled drugs. Yet, controversy persists over whether gabapentin should be similarly classified, underscoring the growing issue of misuse (9). In 2010, the Swedish adverse event reporting system first noted a rise in pregabalin misuse (10). Epidemiological studies show that in countries like the UK, prescriptions for pregabalin and gabapentin increased by 150% and 350%, respectively, between 2012 and 2017 (11, 12). Furthermore, Research suggests that pregabalin has a higher misuse potential than gabapentin due to its faster absorption and quicker onset of action (13). In one case report, a 38-year-old man consumed 8.4 grams of pregabalin daily to achieve euphoria and increased energy, experiencing severe withdrawal symptoms—sweating, anxiety, tachycardia, auditory hallucinations, and suicidal thoughts—within 36 hours of stopping (14). Another case involved an opioid-dependent individual who, unable to obtain dextropropoxyphene, started abusing pregabalin at doses of 10,000 to 12,000 mg/day to manage withdrawal symptoms (15).

In the case presented here, the patient admitted during the medical history review that he was unaware of pregabalin’s potential for misuse and dependence when he began using it under the influence of friends. Among substance misusers, lack of awareness and a dismissive attitude toward drug risks can reduce understanding of misuse consequences, leading to experimentation (16). This behavior can result in dependence, cognitive impairment, emotional instability, and negative effects on social relationships, academic performance, and career prospects, thereby creating a vicious cycle (17–20).

### 3.2 Diagnosis of pregabalin-induced drug dependence

Based on the patient’s clinical presentation, five key diagnostic criteria were met over the past year: (1) A strong craving for pregabalin, even with awareness of its harmful consequences, with the inability to control the urge to continue or increase the dose; (2) Inability to control the frequency or dosage of pregabalin use; (3) Tolerance, requiring higher doses to achieve the same effects; (4) Withdrawal symptoms when reducing or discontinuing use, which could be alleviated by taking the drug again; (5) Significant impairment of daily life and social functioning, including worsening family conflicts, inability to maintain academic performance, and deteriorating health. Based on these criteria and the ICD-10 guidelines, the patient was diagnosed with “multiple drug and other psychoactive substance dependence syndrome.”

The patient’s strong craving for pregabalin was crucial to diagnosing dependence. Initially influenced by friends, he quickly escalated his dosage to achieve a stronger euphoric effect, a typical pattern in drug dependence known as positive reinforcement. He also experienced withdrawal symptoms, such as irritability, insomnia, and depression, when he stopped using the drug, illustrating negative reinforcement where the discomfort of withdrawal drives further use.

### 3.3 Withdrawal symptoms from pregabalin-induced dependence

In this case, the patient experienced physical withdrawal symptoms like palpitations, fatigue, and mild tremors after stopping pregabalin. He also faced severe emotional instability, including irritability, depression, auditory hallucinations, and paranoid delusions. These withdrawal symptoms are linked to disruptions in neurotransmitter systems following cessation.

Firstly, sudden discontinuation after prolonged high-dose pregabalin use can cause dysregulation of the GABA system (21). Although pregabalin does not act directly on GABA receptors, it indirectly affects the system by inhibiting calcium channels and reducing neuronal excitability (22). Long-term high-dose use may lead to the downregulation of GABA receptors, reducing sensitivity to endogenous GABA. When the drug is stopped, the previously suppressed neuronal activity rebounds, weakening the inhibitory effects of the GABA system and leading to increased brain excitability, resulting in symptoms like anxiety, depression, and hallucinations (23, 24).

Secondly, emotional fluctuations and cognitive impairments may arise from adaptive changes in the nervous system during withdrawal, particularly affecting hippocampal memory function (25). Prolonged pregabalin use can disrupt neuroplasticity in the hippocampus, impairing normal neuronal connections. Upon cessation, the hippocampus may not immediately return to normal function, causing memory loss, reduced attention, and diminished processing capacity (26, 27). The patient’s difficulty recalling parts of his medication history supports this observation.

Additionally, the hyperactivity of the glutamatergic system during withdrawal may contribute to anxiety, agitation, and psychotic symptoms (28). Imbalanced neurotransmitter regulation, particularly an increase in glutamate, raises neuronal excitability. This heightened activity affects emotional regulation, causing abnormal emotional responses like paranoia, anxiety, and panic. These emotional changes can worsen cognitive dysfunction, leading to confusion and anxiety when faced with everyday stressors, possibly resulting in mistrust of others or the environment (29–31).

Hallucinations and delusions during pregabalin withdrawal may also be linked to enhanced activity in the dopaminergic system. Studies suggest that drug misuse and withdrawal can modulate dopamine release in the mesolimbic system (32, 33). During pregabalin withdrawal, rebound overactivation of the dopaminergic system may lead to excessive dopamine release, particularly in areas associated with reward and emotional regulation, such as the nucleus accumbens, substantia nigra, and prefrontal cortex. Long-term misuse causes adaptive changes in these dopamine systems, and sudden cessation may result in a sharp dopamine increase, triggering hallucinations and delusions (34, 35). Moreover, chronic pregabalin misuse may induce structural and functional changes in the brain, along with adaptive shifts in central nervous system circuits, intensifying these symptoms during withdrawal (36).

### 3.4 Treatment and prevention strategies for pregabalin-induced drug dependence

Currently, there is a lack of systematic research and guidelines for treating pregabalin-induced dependence. Substitution therapy remains the primary treatment, usually involving other medications to alleviate withdrawal symptoms and gradually replace pregabalin. In this case, benzodiazepines were used as part of a tapering substitution therapy. Benzodiazepines enhance GABA-A receptor-mediated inhibitory neurotransmission, amplifying the effects of GABA and reducing excessive neuronal excitability, thus easing withdrawal symptoms. In the short term, this approach helps patients transition through the withdrawal phase more smoothly. However, regular assessments are necessary, and tapering must be done gradually to prevent new dependencies.

Recent studies increasingly support using antidepressants, anxiolytics, and antipsychotics to improve emotional and psychotic symptoms during withdrawal (37, 38). In this case, venlafaxine was used to regulate serotonin and norepinephrine levels, improving the patient’s mood. Paliperidone and quetiapine fumarate, two atypical antipsychotics, were also used to control psychotic symptoms, such as hallucinations and delusions, while improving sleep and restoring normal patterns. In addition to medication, psychotherapy plays a crucial role in preventing relapse. Cognitive-behavioral therapy (CBT) and other psychological interventions can help patients develop healthy attitudes toward medication and improve their ability to handle stress.

To prevent pregabalin misuse and dependence, a comprehensive approach is needed. First, prescription control should be strictly enforced to ensure that the drug is only used for clear medical purposes. Limiting the dosage and duration of treatment, along with regularly monitoring patients’ medication use, is essential to identify signs of dependence early. For individuals with a history of drug misuse or those at high risk for dependence, prescribing pregabalin should be avoided whenever possible, or the risks and benefits of its use should be carefully evaluated. Additionally, healthcare providers should educate patients on the drug’s mechanisms of action, as well as the potential risks of dependence. Patients should be informed about the consequences of drug misuse and taught to recognize early signs of dependence, such as strong cravings for the drug, increased frequency of use, or using doses beyond the prescribed amount. If misuse is detected, patients should seek medical attention immediately. Early intervention is crucial, and for those already exhibiting signs of dependence, referral to specialized dependence treatment centers is recommended. These centers can provide professional treatment for drug dependence, along with psychological therapy and behavioral interventions, to help patients restore normal life.

In conclusion, treating pregabalin dependence requires a comprehensive approach that takes into account the drug’s pharmacological properties, individual differences, and the role of social support systems. Further research is needed to understand the mechanisms behind pregabalin misuse and develop better prevention and intervention strategies. Increasing awareness and improving the management of pregabalin misuse will remain key priorities in the field of dependence prevention.

## Data Availability

The original contributions presented in the study are included in the article/**Supplementary Material**. Further inquiries can be directed to the corresponding author.

## References

[1] McHugh RK Nielsen S Weiss RD . Prescription drug abuse: from epidemiology to public policy. J Subst Abuse Treat. (2014) 48:1–7. doi: 10.1016/j.jsat.2014.08.004 25239857 PMC4250400

[2] Pittenger C Desan PH . Gabapentin abuse, and delirium tremens upon gabapentin withdrawal. J Clin Psychiatry. (2007) 68:483–4. doi: 10.4088/jcp.v68n0320a 17388722

[3] Evoy KE Sadrameli S Contreras J Covvey JR Peckham AM Morrison MD . Abuse and misuse of pregabalin and gabapentin: A systematic review update. Drugs. (2020) 81:125–56. doi: 10.1007/s40265-020-01432-7 33215352

[4] Hägg S Jönsson AK Ahlner J . Current evidence on abuse and misuse of gabapentinoids. Drug Saf. (2020) 43:1235–54. doi: 10.1007/s40264-020-00985-6 PMC7686181 32857333

[5] Blommel ML Blommel AL . Pregabalin: An antiepileptic agent useful for neuropathic pain. Am J Health Syst Pharm. (2007) 64:1475–82. doi: 10.2146/ajhp060371 17617497

[6] Schifano F Chiappini S . Pregabalin: A range of misuse-related unanswered questions. CNS Neurosci Ther. (2019) 25:659–60. doi: 10.1111/cns.13115 PMC6488882 30834646

[7] Chiappini S Schifano F . What about "Pharming"? Issues regarding the misuse of prescription and over-the-counter drugs. Brain Sci. (2020) 10:736. doi: 10.3390/brainsci10100736 33066476 PMC7602178

[8] Chiappini S Schifano F . A decade of gabapentinoid misuse: An analysis of the European Medicines Agency's 'Suspected Adverse Drug Reactions' database. CNS Drugs. (2016) 30:647–54. doi: 10.1007/s40263-016-0359-y 27312320

[9] Peckham AM Ananickal M Sclar D . Gabapentin use, abuse, and the US opioid epidemic: the case for reclassification as a controlled substance and the need for pharmacovigilance. RMHP Volume. (2018) 11:109–16. doi: 10.2147/rmhp.s168504 PMC6103607 30154674

[10] Schwan S Sundström A Stjernberg E Hallberg E Hallberg P . A signal for an abuse liability for pregabalin—results from the Swedish spontaneous adverse drug reaction reporting system. Eur J Clin Pharmacol. (2010) 66:947–53. doi: 10.1007/s00228-010-0853-y 20563568

[11] Spence D . Bad medicine: gabapentin and pregabalin. BMJ. (2013) 347:f6747–7. doi: 10.1136/bmj.f6747 24212252

[12] Morrison E Sandilands E Webb D . Gabapentin and pregabalin: do the benefits outweigh the harms? J R Coll Physicians Edinb. (2017) 47:310–3. doi: 10.4997/jrcpe.2017.402 29537399

[13] Driot D Jouanjus E Oustric S Dupouy J Lapeyre-Mestre M . Patterns of gabapentin and pregabalin use and misuse: Results of a population-based cohort study in France. Br J Clin Pharmacol. (2019) 85:1260–9. doi: 10.1111/bcp.13892 PMC6533441 30737829

[14] Nordgaard J Jürgens G . Pregabalin can cause addiction and withdrawal symptoms. Ugeskrift læger. (2015) 177:38–9. 25612958

[15] Chaudhury S Sahu S Kumar S Saldanha D . A case of pregabalin addiction. Ind Psychiatry J. (2021) 30:352–2. doi: 10.4103/0972-6748.328855 PMC8611539 34908735

[16] Motyka MA Al-Imam A . Representations of psychoactive drugs' use in mass culture and their impact on audiences. Int J Environ Res Public Health. (2021) 18:6000. doi: 10.3390/ijerph18116000 34204970 PMC8199904

[17] Favrod-Coune T Broers B . The health effect of psychostimulants: A literature review. Pharmaceutics. (2010) 3:2333–61. doi: 10.3390/ph3072333 PMC4036656 27713356

[18] Liang R Wang L Sun S Zheng C Yang J Ming D . Medial prefrontal cortex and hippocampus in mice differently affected by simulated microgravity and social isolation associated with the alternation of emotional and cognitive functions. Life Sci Space Res. (2022) 33:21–32. doi: 10.1016/j.lssr.2022.02.001 35491026

[19] Vink JM Schellekens A . Relating addiction and psychiatric disorders. Science. (2018) 361:1323–4. doi: 10.1126/science.aav3928 30262491

[20] Chen A Mari S Grech S Levitt J . What we know about massively multiplayer online role-playing games. Harv. Rev Psychiatry. (2020) 28:107–12. doi: 10.1097/HRP.0000000000000247 32134835

[21] Alles SRA Cain SM Snutch TP . Pregabalin as a pain therapeutic: beyond calcium channels. Front Cell Neurosci. (2020) 14:83. doi: 10.3389/fncel.2020.00083 32351366 PMC7174704

[22] Schifano F Chiappini S Corkery JM Guirguis A . Abuse of prescription drugs in the context of novel psychoactive substances (NPS): A systematic review. Brain Sci. (2018) 8:73. doi: 10.3390/brainsci8040073 29690558 PMC5924409

[23] Zaccara G Gangemi P Perucca P Specchio L . The adverse event profile of pregabalin: A systematic review and meta-analysis of randomized controlled trials. Epilepsia. (2011) 52:826–36. doi: 10.1111/j.1528-1167.2010.02966.x 21320112

[24] Gundogmus İ Karagöz A Algül A . First-episode psychosis induced by pregabalin withdrawal: a case report. Psychiatry Clin Psychopharmacol - PCP. (2018) 28:461–3. doi: 10.1080/24750573.2018.1452523

[25] Behroozi Z Jafarpour M Razmgir M Saffarpour S Azizi H Kheirandish A . The effect of gabapentin and pregabalin administration on memory in clinical and preclinical studies: a meta-analysis and systematic review. BMC Psychiatry. (2023) 23:1–14. doi: 10.1186/s12888-023-04696-x 37069609 PMC10111701

[26] Sałat K Gdula-Argasińska J Malikowska N Podkowa A Lipkowska A Librowski T . Effect of pregabalin on contextual memory deficits and inflammatory state-related protein expression in streptozotocin-induced diabetic mice. Naunyn-Schmiedeberg’s Arch Pharmacol. (2016) 389:613–23. doi: 10.1007/s00210-016-1230-x PMC4866991 26984821

[27] Wu W-F Chen C Lin J-T Jiao X-H Dong W Wan J . Impaired synaptic plasticity and decreased glutamatergic neuron excitability induced by SIRT1/BDNF downregulation in the hippocampal CA1 region are involved in postoperative cognitive dysfunction. Cell Mol Biol Lett. (2024) 29. doi: 10.1186/s11658-024-00595-5 PMC11112897 38783169

[28] Nasir M Trujillo D Levine J Dwyer JB Rupp ZW Bloch MH . Glutamate systems in DSM-5 anxiety disorders: their role and a review of glutamate and GABA psychopharmacology. Front Psychiatry. (2020) 11:548505. doi: 10.3389/fpsyt.2020.548505 33329087 PMC7710541

[29] Bialer M Johannessen SI Kupferberg HJ Levy RH Perucca E Tomson T . Progress report on new antiepileptic drugs: A summary of the Eigth Eilat Conference (EILAT VIII). Epilepsy Res. (2006) 73:1–52. doi: 10.1016/j.eplepsyres.2006.10.008 17158031

[30] Javitt D . Glutamatergic theories of schizophrenia. Israel J Psychiatry related Sci. (2010) 47 1:4–16. 20686195

[31] Lerner A Klein M . Dependence, withdrawal and rebound of CNS drugs: an update and regulatory considerations for new drugs development. Brain. Commun. (2019) 1. doi: 10.1093/braincomms/fcz025 PMC7425303 32954266

[32] Nestler EJ . Transcriptional mechanisms of drug addiction. Clin Psychopharmacol Neurosci. (2012) 10:136–43. doi: 10.9758/cpn.2012.10.3.136 PMC3569166 23430970

[33] Koob GF Volkow ND . Neurobiology of addiction: a neurocircuitry analysis. Lancet Psychiatry. (2016) 3:760–73. doi: 10.1016/s2215-0366(16)00104-8 PMC6135092 27475769

[34] Barrett J Kittler L Singarajah C . Acute pregabalin withdrawal: a case report and review of the literature. Southwest J Pulmonary Crit Care. (2015) 10:306–10. doi: 10.13175/swjpcc059-15

[35] Taha SHN Zaghloul HS Ali AAER Gaballah IF Rashed LA Aboulhoda BE . The neurotoxic effect of long-term use of high-dose Pregabalin and the role of alpha tocopherol in amelioration: implication of MAPK signaling with oxidative stress and apoptosis. Naunyn-Schmiedeberg’s Arch Pharmacol. (2020) 393:1635–48. doi: 10.1007/s00210-020-01875-5 32377769

[36] Schifano F . Misuse and abuse of pregabalin and gabapentin: cause for concern? CNS Drugs. (2014) 28:491–6. doi: 10.1007/s40263-014-0164-4 24760436

[37] Brandt L Bschor T Henssler J Müller M Hasan A Heinz A . Antipsychotic withdrawal symptoms: A systematic review and meta-analysis. Front Psychiatry. (2020) 11:569912. doi: 10.3389/fpsyt.2020.569912 33132934 PMC7552943

[38] Van Leeuwen E van Driel ML Horowitz MA Kendrick T Donald M De Sutter AI . Approaches for discontinuation versus continuation of long-term antidepressant use for depressive and anxiety disorders in adults. Cochrane Database Syst Rev. (2021) 2021:CD013495. doi: 10.1002/14651858.cd013495.pub2 PMC8092632 33886130

