# Supplementary material for: A case report of pregabalin misuse leading to drug dependence
Source: Front Psychiatry. 2025 Mar 21;16:1511168. doi: 10.3389/fpsyt.2025.1511168 (PMC11969338; doi:10.3389/fpsyt.2025.1511168)
Supplement: Supplementary file 1 [file Table1.docx]

Table S1. Changes in the patient's symptoms and signs and adjustments to the treatment plan after admission.

| **Date** | **Symptoms and Signs** | **Treatment** | |
| --- | --- | --- | --- |
| Day 1 | The HAMD score was 16, the HAMA score was 25, and the PANSS score was 98. | Diazepam 10mg Q12h ivgtt  Venlafaxine 75mg Qd po  Paliperidone 3mg Qd po |  |
| Day 3 | The patient reported having not slept all night and continued to feel low in mood and irritable. The patient denied experiencing hallucinations or delusions.  The HAMD score was 15, the HAMA score was 24, and the PANSS score was 71. | Increase venlafaxine to 150 mg Qd  Add quetiapine fumarate 0.1g Qn po |  |
| Day 7 | The patient reported improved sleep at night but experienced daytime fatigue. Mood had improved compared to before, with no significant irritability or restlessness observed.  The HAMD score was 13, the HAMA score was 15, and the PANSS score was 43. | Decrease diazepam to 10mg Qn  Increase venlafaxine to 225 mg Qd |  |
| Day 10 | The patient reported no significant palpitations, fatigue, or hand tremors but still expressed a desire to take pregabalin. | Discontinue diazepam  Add oxazepam 30mg Qn po |  |
| Day 15 | The patient's withdrawal symptoms had essentially resolved, and the craving for the medication had decreased.  The HAMD score was 6, and the HAMA score was 10. | Discontinue oxazepam |  |
| Day 21 | The patient reported that sleep was mostly normal, with no significant withdrawal symptoms, and no strong cravings for the medication. Mood was stable.  The HAMD score was 3, the HAMA score was 7, and the patient expressed a desire to be discharged. | Discharge medication:  Venlafaxine 225mg Qd po  Paliperidone 3mg Qd po |  |
